# Menstrual Cycle Changes in Vagally-Mediated Heart Rate Variability Are Associated with Progesterone: Evidence from Two Within-Person Studies

**DOI:** 10.3390/jcm9030617

**Published:** 2020-02-25

**Authors:** Katja M. Schmalenberger, Tory A. Eisenlohr-Moul, Marc N. Jarczok, Monika Eckstein, Ekaterina Schneider, Ines G. Brenner, Kathleen Duffy, Sophie Schweizer, Jeff Kiesner, Julian F. Thayer, Beate Ditzen

**Affiliations:** 1 Institute of Medical Psychology, Center for Psychosocial Medicine, University Hospital Heidelberg, Ruprecht-Karls University Heidelberg, 69115 Heidelberg, Germany; monika.eckstein@med.uni-heidelberg.de (M.E.); ekaterina.schneider@med.uni-heidelberg.de (E.S.); ines.brenner11@gmail.com (I.G.B.); sophie.schweizer@med.uni-heidelberg.de (S.S.); 2 Women’s Mental Health Research Program, Department of Psychiatry, University of Illinois at Chicago, Chicago, IL 60612, USA; t.eisenlohr.moul@gmail.com (T.A.E.-M.); kduffy7@luc.edu (K.D.); 3 Clinic for Psychosomatic Medicine and Psychotherapy, Ulm University Medical Center, 89081 Ulm, Germany; marc.jarczok@uniklinik-ulm.de; 4 Department of General Psychology, University of Padua, 35131 Padua, Italy; jeff.kiesner@unipd.it; 5 Department of Psychological Science, School of Social Ecology, University of California Irvine, Irvine, CA 92697-7085, USA; jfthayer@uci.edu

**Keywords:** menstrual cycle, progesterone, estradiol, estrogen, ovarian hormones, heart rate variability, cardiac vagal activity, cardiac vagal tone, autonomic nervous system

## Abstract

A recent meta-analysis revealed that cardiac vagal activity (mostly indicated by vagally-mediated heart rate variability; HRV) decreases significantly from the follicular to luteal menstrual cycle phase in naturally-cycling participants. However, the question remains as to whether cyclical changes in estradiol (E2), progesterone (P4), or both are responsible for HRV fluctuations. We present the first studies to use repeated measures of E2, P4, and HRV across the cycle to model both the unique and interactive effects of person-centered E2 and P4 on HRV in multilevel models. In study one, 40 naturally-cycling participants were assessed weekly across four weeks, and were blind to the cycle focus of the study. In study two, 50 naturally-cycling participants were examined in three precisely defined cycle phases via ovulation testing. Both studies revealed that only P4 was correlated with HRV, such that higher-than-usual P4 significantly predicted lower-than-usual HRV within a given participant. In line with this, cycle phase comparisons revealed lower HRV in the mid-luteal phase (characterized by elevated P4) than in other phases. No significant main or interactive effects of E2 on HRV were found. Future female health studies should investigate individual differences in these effects and potential consequences of cyclical HRV changes on daily functioning.

## 1. Introduction

Cardiac vagal activity describes the restorative parasympathetic innervation of the heart, which is exercised by the vagus nerve (tenth cranial nerve). In recent years, numerous studies have related decreased levels of cardiac vagal activity to lower emotional and cognitive self-regulatory capacity [1,2,3,4,5] and a higher risk for various physical diseases (e.g., [6,7,8,9,10]) which resulted in cardiac vagal activity emerging as a transdiagnostic biomarker for mental and physical health [11,12,13]. The most commonly used indicator for cardiac vagal activity is the vagally-mediated components of heart rate variability (HRV). HRV results from the interplay of the parasympathetic and the sympathetic branch of the autonomic nervous system (ANS) and describes the variability in the time span between successive heartbeats as a function of current internal and external demands to support the organism in an optimal response to them. Most research on vagally-mediated HRV has focused on HRV in the respiratory frequency range, also known as respiratory sinus arrhythmia (RSA). RSA describes the degree to which the heart rate changes between inhaling and exhaling air from the lungs, which shows no sensitivity to sympathetic nervous system activity but is mediated by the vagal nerve. This has led to the use of tonic RSA levels as a proxy for vagally-mediated HRV [14].

Given the role of vagally-mediated HRV as a biomarker for physical and mental health, understanding how HRV varies between and within person is critical. One factor contributing to inter-individual differences in HRV is psychopathology. For example, a recent meta-analysis [15] suggests significant HRV reductions in patients with borderline personality disorder (i.e., the most common personality disorder in clinical settings [16]; BPD) relative to healthy controls. Key features of the disorder like emotional lability and impulsivity [17] represent impaired emotional self-regulatory capacity and reduced inhibitory control which, in turn, have been linked to lower vagally-mediated HRV in numerous studies (e.g., [2,18,19,20]). Regarding factors giving rise to intra-individual changes in HRV, one of these could be the natural menstrual cycle with its systematic fluctuations of the ovarian hormones estradiol (E2) and progesterone (P4). From the onset of menses until the end of ovulation (i.e., follicular phase), both E2 and P4 levels are relatively low with a swift increase and primary peak of E2 shortly before ovulation. In the timespan after ovulation until the day before the subsequent menstrual onset (i.e., luteal phase), E2 levels first plummet but remain generally higher compared to the follicular phase while P4 levels begin to gradually increase. After a primary peak of P4 and a secondary peak of E2 levels, both E2 and P4 withdraw premenstrually and a new cycle begins. Longitudinal and experimental work shows that the menstrual cycle can modulate affective and behavioral outcomes (e.g., [21,22,23,24]) as well as physiological functions, like nutritional metabolism (e.g., [25,26]) and vagally-mediated HRV (e.g., [27]). Intra-individual variations in vagally-mediated HRV across the menstrual cycle with their potential implications for female daily functioning and well-being have therefore been the subject of various empirical studies. Our recent systematic review and meta-analysis of repeated measures studies demonstrated that cardiac vagal activity (indicated by vagally-mediated HRV in most of the included studies) varies significantly across the natural menstrual cycle [28], showing a significant decrease from the follicular to the luteal phase (*d* = −0.39; conventionally medium effect size [29]). The present paper builds on this recent meta-analysis by reporting on two studies examining the unique roles of E2 and P4 fluctuations in cardiac vagal activity across the cycle.

To date, only seven studies (of roughly 49 currently available studies on cardiac vagal activity across the menstrual cycle as suggested by our meta-analysis [28] and a recent review [30]) have reported associations between ovarian hormone levels and cardiac vagal activity, with mixed results [27,31,32,33,34,35,36]. However, four major methodological and statistical limitations in these studies prevent clear conclusions. First, many of these studies correlate absolute hormone levels with absolute cardiac vagal activity (using the full repeated-measures dataset), which fails to isolate a within-person variance in ovarian hormones as the critical predictor (by “person-centering” the repeated hormonal predictors [37]); instead, using a variable that dilutes the within-person variance in hormone levels across the cycle with the between-person (or, more probably, between-cycle) variance in hormone levels [31,32,33,35]. Second, the use of a repeated-measures dataset in a simple correlation analysis violates the statistical assumption that observations are independent from one another (since repeated observations are nested within participants). Third, some studies appear to subset their repeated measures datasets by cycle phase, then perform correlations between absolute hormone levels and cardiac vagal activity (in a between-person manner); this fails to capture the predictor variance of interest, which is that of within-person changes in hormones across the cycle [27,34]. Finally, each of these studies appeared to examine E2 and P4 as predictors of cardiac vagal activity in separate models, which fails to test both the unique effects of changes in each hormone and the potential interactive effects of these hormones [27,31,32,33,34,35,36].

The aim of this work is to use repeated measures studies to examine how E2 and P4 are associated with HRV within a given woman in the context of the natural menstrual cycle. Here, we present two longitudinal studies each following participants across the cycle and obtaining repeated measures of E2, P4, and vagally-mediated, resting state HRV. By using within-person designs and examining unique and interactive effects of E2 and P4 on HRV, both studies improve upon prior methods. Although both use repeated measures of hormones and HRV across the cycle, they also differ in participant blinding and visit timing, which provides an opportunity for conceptual replication (demonstration that an effect does not depend on the use of one specific methodology [38]). In each study, we first attempted to directly replicate our recent meta-analytic finding that menstrual cycle phase influences HRV [28], then examine our primary question regarding associations of cyclical ovarian hormones with HRV.

### Hypotheses

A large meta-analysis based on over 1000 participants revealed a significant decrease in vagally-mediated HRV from the follicular to the luteal cycle phase [28]. At the hormonal level, these two cycle phases differ mainly regarding P4 levels (low P4 in the follicular phase and high P4 in the luteal phase). Based on these findings, we constructed the following hypotheses:

1. Vagally-mediated HRV is lower in the midluteal phase relative to mid-follicular and ovulatory phases.
2. Within-person fluctuations of vagally-mediated HRV across the cycle are more strongly associated with P4 than with E2.
3. Higher levels of P4 are associated with lower levels of vagally-mediated HRV.

## 2. Study One (United States)

### 2.1. Materials and Methods

#### 2.1.1. Overview and Study Design

The primary purpose of the first study, conducted in America, was to examine correlations of hormones with weekly dimensional symptoms of BPD in college students [39]. In this sample, hormones and HRV were measured on the same weekday for four weeks in a row, irrespective of cycle phase. Participants were blinded to the focus of the study, reducing any impact of biased or inaccurate beliefs (about premenstrual changes in function [40,41]) on psychological states associated with HRV (e.g., effort, stress [42]). Although this blinding reduces the precision of cycle phase estimations, it allows for clear examination of how hormones and HRV covary within a given individual across the cycle.

#### 2.1.2. Participants

Naturally-cycling college students were recruited from introductory psychology courses for a study on “Health and Daily Experiences” (with no mention of the cycle) in 2012-2013. Exclusion criteria were (1) any deviation from the natural monthly menstrual cycle (e.g., use of hormonal contraceptives, amenorrhea, pregnancy, etc.). (2) use of psychopharmacological drugs, (3) lack of English fluency, (4) menstrual cycle length lower than 25 or greater than 35 days, and/or (5) past or present diagnosis of a psychotic disorder (excluding dissociation). 

To ensure response variability across a range of BPD symptoms, eligible participants were recruited equally across four symptom ranges based on their responses on the Personality Assessment Inventory–Borderline subscale (PAI-BOR [43]) during a classwide screening (*N* = 439): low average (T < 50), high average (50 < T < 60), above average (60 < T < 70), and high (T > 70). The final sample (*N* = 40) averaged 18.69 years old (*SD* = 1.42). At the end of the study, the BPD module of the Structured Clinical Interview for Diagnosis–II (SCID-II [44]) was administered to determine diagnostic status and number of DSM-5 BPD criteria met. Of the 40 participants, 22 met zero criteria, 13 met between 1 and 4 criteria, and 5 met full DSM-5 criteria for BPD. Thirty-four participants identified as Caucasian, 4 as African American, and 2 as Asian; three of those identifying as Caucasian also identified as Hispanic ethnicity. All participants identified as heterosexual. Self-reported height and weight were used to calculate BMI (*M* = 23.13; *SD* = 3.64).

#### 2.1.3. HRV Assessment

At each of the four weekly visits, a six-minute, seated, baseline electrocardiogram (ECG) was sampled at a rate of 1000 Hz applying a lead II configuration using a BioNex system from MindWare Technologies LTD (Westerville, OH, USA) and amplified utilizing the appropriate module. Time of day varied across participants; however, it was identical within a given participant over time, mitigating the influence of diurnal variance on our within-person associations. Instructions for the recording were as follows: “Now we are going to gather a baseline “snapshot” of your heart’s activity. Please sit quietly for approximately 5 min while the machine records your readings. During this baseline period, please try to avoid moving around; you don’t have to remain totally still, but avoiding a lot of unnecessary movement will help the signal to stay clear. You can think about anything you like during this time”. The final five minutes of the recoding were utilized for data preparation and analysis using MindWare HRV 3.0.1 software (MindWare Technologies LTD; Westerville, OH, USA). Spectral analysis of ECG data was used to quantify high frequency (HF) heart rate variability (0.15−0.40 Hz), which serves as a frequency domain measure of RSA, separately across the final five 60-s epochs. Correction of movement artifacts as well as errors in computerized marking of R-peaks was accomplished in MindWare HRV 3.0.1 software, which utilized an algorithm to mark statistically improbable R peaks for manual inspection. These were inspected and adjusted according to standards suggested by the Task Force of the European Society of Cardiology and the North American Society of Pacing and Electrophysiology [45]. Approximately 0.053% of R peaks (beats) were adjusted during this inspection and cleaning process due to movement, computer error in specification of the R peak, or physiologically abnormal beats. Once each epoch was cleaned, the average high frequency (HF) HRV was calculated for each visit across the five one-minute epochs, and a log transformation was applied (resulting in a final variable referred to as HFlog). HRV values were missing for 4 observations due to sample recording failures.

#### 2.1.4. Hormone Analyses

Participants were asked to avoid the following in the 12 h before the lab: chewing gum, smoking, alcohol, more than one caffeinated beverage, and over-the-counter, prescription, or recreational drugs. At some visits, participants reported past-12-h smoking (13 visits), drinking more than 1 caffeinated beverage (2 visits), and using over-the-counter drugs (7 visits). Covarying these violations did not alter the results. Participants provided 1.8 mL saliva via passive drool through a straw into a polypropylene vial (Salimetrics; State College, PA, USA) at each visit. Samples were kept at −20 °C in a chest freezer until assay for E2 and P4 using ELISA kits (Salimetrics). Intra-assay coefficient of variation was 1.6% for E2 and 2.7% for P4; inter-assay coefficient of variation was 2.2% for E2 and 5.4% for P4. Hormonal values were missing at 2 visits due to insufficient saliva sampling. 

#### 2.1.5. Procedure

After screening for eligibility, participants were invited to schedule five consecutive weekly visits at the same day and time each week between 7:30 am and 7:30 pm (four assessment visits, one brief SCID-II visit). Each visit took place individually in a small room with a computer in front of the participant. Visits started with the baseline ECG and collection of saliva. Subsequently, participants completed questionnaires related to past-week BPD symptoms [39]. The overall length of the visit was about 60 min. Of the 160 visits (40 participants with 4 laboratory sessions each), 125 could be completed as originally scheduled, 29 had to be rescheduled, but could be completed within 3 days of the missed appointment, and 6 had to be postponed to the week following the last originally scheduled visit. Participation was reimbursed via course credit. The study was approved by the Institutional Review Board of the University of Kentucky (approval code: 12-0632).

#### 2.1.6. Cycle Phase Criteria

Precise assignment of assessments to well-defined cycle phases is essential for obtaining meaningful results from menstrual cycle studies [28]. As described above, in order to disguise the menstrual cycle focus of the study, visits were not scheduled to coincide with a specific cycle phase. Instead, participants reported on pre-study and post-study menses start dates, as well as reporting daily menstrual bleeding during study participation. Consequently, two approaches for assigning the assessments to one of the four cycle phases (mid-luteal, perimenstrual, mid-follicular, ovulatory) were available: (1) The forward-count cycle day approach which assigns each cycle day a number based on the last menstrual onset (i.e., day +1) by counting forward, and (2) the backward-count cycle day approach which assigns each cycle day a number based on the subsequent menstrual onset (where day 1 is menstrual onset and there is no day 0), by counting backward from that day. The day before the next menstrual onset is day −1 and so forth. Due to the relative robust luteal phase length of 14 days (from ovulation to the next menstrual onset), this method is particularly suitable for determining the ovulatory and mid-luteal phase [28].

For each visit, we used a combination of forward- and backward-count methods to identify the cycle day on which it took place in retrospect. The following cycle phase criteria were applied: ovulatory was coded as cycle day −12 to −17; mid-luteal was coded as cycle day −4 to −11; perimenstrual was coded as cycle day −3 to +3 (where day 1 is menstrual onset and there is no day 0); and mid-follicular was coded as cycle day +5 to +10. In 55 cases, neither the forward nor the backward count fell within these windows; these observations were excluded to increase precision of our estimates. Therefore, 105 of the visit observations were coded as belonging to one of the four phase categories described above.

#### 2.1.7. Statistical Analyses

Using SAS 9.4, we utilized PROC MIXED to examine both cycle phase and hormone effects using two-level multilevel models, with visits (Level 1) nested within participants (Level 2); in each model, a random intercept (with an unstructured covariance structure) was included to account for between-person differences in mean HFlog, an autoregressive term was included to account for serial correlation across sessions, session number was included as a covariate to control for order effects. A restricted maximum likelihood approach was utilized, and the denominator degrees of freedom were calculated using the Kenward-Roger method. To test cycle phase predictions (i.e., Hypothesis 1), we tested a model predicting HFlog from session number and a categorical cycle phase predictor to examine all pairwise comparisons between cycle phases. With four observations per person, it was not possible to additionally estimate random effects separately for each of the three phase contrasts. To test hormone-related predictions (i.e., Hypotheses 2 and 3), we examined a model predicting HFlog from session number, person-centered E2, person-centered P4, and their interaction. Random effects of hormonal predictors were also included where model convergence allowed. The corresponding dataset for study one is provided in the Supplementary Materials 1.

### 2.2. Results

#### 2.2.1. Descriptive Analyses

For cycle phase analyses, a total of 105 visits from 38 participants were included. Descriptive information for E2, P4 and HFlog levels are listed for each cycle phase separately in Table 1. Although E2 demonstrated the highest absolute level in the ovulatory phase, this increase was not exponentially higher than the other phases. This is consistent with our cycle day count-based method of determining cycle phase, which includes both peak E2 days as well as lower-E2 peri-ovulatory days. P4 was highest in the midluteal phase.

For hormone analyses, a total of 158 visits from 40 participants could be included.

#### 2.2.2. Multilevel Regression Models Predicting HFlog from Menstrual Cycle Phase

Results of multilevel models examining pairwise comparisons of HFlog between each cycle phase are presented in Table 2. As predicted in Hypothesis 1, HFlog was lower in the midluteal phase than in both follicular assessments (i.e., mid-follicular and ovulatory assessments). Midluteal HFlog was also significantly lower than perimenstrual HFlog. No other cycle phase comparisons were significant.

#### 2.2.3. Multilevel Regression Models Predicting HFlog from E2 and P4

Results of the model predicting HFlog from E2, P4, and their interaction are reported in Table 3. A significant random slope was observed for P4, indicating significant individual differences in the degree to which changes in P4 were associated with changes in HFlog. As predicted, only P4 was significantly associated with lower HFlog. Hypotheses 2 and 3 could thus be confirmed. Of note, this is consistent with the finding that the midluteal phase, which is characterized by a peak in P4, shows the lowest HFlog.

## 3. Study Two (Germany)

### 3.1. Materials and Methods

#### 3.1.1. Overview and Study Design

The primary purpose of the second study, conducted in Germany, was to examine the covariation of salivary ovarian hormones with HRV in healthy community volunteers. Fifty participants completed an initial visit as well as three assessment visits in different cycle phases representing specific hormonal profiles (perimenstrual: falling and low E2 and P4; ovulatory: high E2, low P4; mid-luteal: high E2 and P4). At each visit, baseline HRV and salivary E2 and P4 levels were assessed. Participants were generally healthy, not sampled for any specific personality trait and knew about the menstrual cycle focus of the study.

#### 3.1.2. Participants

Participants were recruited from the community in Heidelberg, Germany, for a study of the “biology of female decision making”, that investigated “which physical factors influence everyday decisions” and “the role daily stress plays in them”. Data collection was conducted in 2018. To be included in the study, one had to be 18–45 years old. Exclusion criteria were identical to those above, with the exceptions that participants needed to be fluent in German rather than English, and needed to have a BMI between 18 and 26.

Of the 86 individuals who expressed interest, 67 completed a phone screen for eligibility. Fifty-three of those were eligible and invited to an individual introductory meeting, while 9 did not meet eligibility criteria (*n* = 3 taking antidepressants, *n* = 2 starting oral contraceptives, *n* = 3 with cycle irregularities, *n* = 1 with pacemaker) and 5 declined. Over the course of the study, 3 participants had to be excluded due to cycle irregularities: One participant showed two consecutive anovulatory cycles (i.e., her ovulation tests did not yield a positive result); the other two participants’ luteal phases were very short (i.e., the time span from the day after the positive ovulation test to the day before the onset of the subsequent menstrual onset was less than 6 days).

The final sample consisted of 50 participants with a mean age of 24.8 years (*SD* = 5.8). The majority of the participants (82%) were university students in undergraduate or graduate programs. 86% of participants identified as heterosexual, 6% identified as homosexual, 4% identified as bisexual and 4% did not specify their sexual orientation. 

#### 3.1.3. Ovulation Testing

Ovulation can be determined by testing the urinary levels of the luteinizing hormone (LH) which shows one singular peak per cycle at ovulation. Urine-based LH tests by PURBAY^®^ (Münster, Germany) with a sensitivity of 10 mIU/mL were used to identify the day of the LH surge. During their introductory visit, participants were trained to use the tests and instructed to use their daily test at the same time each day. Participants notified the study team when a positive test appeared, and were encouraged to send a photo of the test to the study team if they were unsure about the result.

#### 3.1.4. HRV Assessment

A ten-minute, seated, baseline ECG was sampled at a rate of 1000 Hz using AcqKnowledge^®^ 5 from Biopac Systems Inc (Goleta, CA, USA) and amplified utilizing the appropriate module (ECG100C). Time of day of the recording varied across participants; however, it was kept as identical as possible within a given participant across all three visits. Participants were given the following instructions prior to the recording: “Now we are going to gather a baseline recording of your body’s activity. Please sit quietly and try to avoid moving around for the next 10 min”. Throughout the recording, participants were sitting alone in front of a computer with the monitor turned off. Due to a mechanical failure in the ECG recording device, no HRV data was available for one participant during one of her three visits.

Data were prepared and cleaned for analysis using Kubios HRV Premium 3.2.0 software (Kubios Oy; Kuopio, Finland). As in study one, spectral analysis of ECG data was used to quantify HF heart rate variability (0.15–0.40 Hz), which serves as a frequency domain measure of RSA, separately across two 5-min epochs. Correction of artifacts as well as errors in computerized marking of R-peaks was accomplished with the Kubios software’s automatic artifact correction. Subsequently, computerized marking of R-peaks were manually inspected and adjusted according to standards suggested by the Task Force of the European Society of Cardiology and the North American Society of Pacing and Electrophysiology [45]. Approximately 0.518% of R peaks (beats) were adjusted during this inspection and cleaning process due to movement, computer error in specification of the R peak, or physiologically abnormal beats. Once each epoch was cleaned, the average high frequency (HF) HRV was calculated for each visit across the two five-minute epochs and a log transformation was applied analogue to study one (resulting in a final variable that is referred to as HFlog).

#### 3.1.5. Hormone Analyses

During enrollment and the day before each visit, participants were personally instructed and reminded via e-mail to abide by the following rules in the 2 h before the visit in order to eliminate possible confounders: no intake of beverages other than water or herbal tea (including caffeine, juice, soft drinks), no intake of food containing protein, no excessive exercise (since this could alter the heart rate and thus the ECG measurement used to analyze HRV) and no smoking. All participants confirmed on arrival at the laboratory that they had adhered to the rules. Additionally, since saliva samples were always taken at the end of the 75-min visit, it was possible to ensure that these rules were adhered to in the 75 min prior to saliva collection. During their visit in the laboratory, water was made available to the participants. However, it was removed 10 min before the end of the session in order to not dilute the saliva shortly before its collection.

Two SaliCaps (IBL; Hamburg, Germany) per visit were employed to collect a total of 3 mL saliva via passive drool through a straw. Samples were kept at −80 °C in an upright freezer until analysis in the in-house laboratory of the Institute of Medical Psychology, Heidelberg. After an initial centrifugation at 3540 rpm for 10 min, the salivary E2 and P4 levels were assessed in duplicates via luminescence immunoassay (IBL). Intra-assay coefficient of variation was 4.8% for E2 and 4.7% for P4; inter-assay coefficient of variation was 5% for E2 and 4% for P4.

#### 3.1.6. Procedure

After the participants had been successfully screened regarding eligibility requirements, they received detailed information about the study and gave their informed consent for participation in an individual introductory meeting. Subsequently, three visits were scheduled in different menstrual cycle phases (i.e., menstrual, ovulatory, mid-luteal). At the participant’s convenience, visits took place at any time between 7.30 am and 7.30 pm, with the time of day being kept as constant as possible across all three visits for each participant. The baseline heart rate was measured upon arrival at the laboratory, after which participants completed five computer-based cognitive tasks (results are not reported here). The saliva sample was taken at the end of the visit. The total length of the visit was about 75 min. After all three visits, participants were invited to a final individual meeting, where they received financial compensation for the time spent of 60 € (80 € if four visits were conducted). The study was approved by the Ethics Committee of the Medical Faculty Heidelberg (University Hospital Heidelberg; approval code: S-322/2017).

#### 3.1.7. Session Scheduling

Each participant was invited to a visit in her menstrual phase (between cycle day +2 to +4, where cycle day +1 is menses onset and there is no day 0), ovulatory phase (on the day of the positive ovulation test (i.e., day 0) or day +1 following the positive ovulation test), and mid-luteal phase (between day +6 to +8 following the positive ovulation test, where day 0 is the day of the positive ovulation test). Hence, sessions were determined individually as a function of self-reported menstrual onset (menstrual assessment) and a positive ovulation test result (ovulatory and mid-luteal assessment). With each participant, it was decided individually on which day in her cycle she would start to perform daily ovulation testing: During her introductory meeting, the participant was asked to indicate the shortest cycle of her previous six menstrual cycles. From this cycle length, 14 days (i.e., the expected length of the luteal phase) were subtracted to determine the cycle day on which ovulation had occurred earliest in the preceding months. The participant was instructed to begin daily ovulation testing five days before this calculated ovulation day to minimize the risk of missing ovulation.

To avoid order effects across the three visits, the order in which the visits were carried out was counterbalanced across participants. Participants who did the perimenstrual visit first completed all three visits within one biological menstrual cycle (i.e., time span from onset of menses to the day before the subsequent onset of menses); other participants completed visits across two biological menstrual cycles. Participants starting with the mid-luteal phase had to carry out ovulation tests in two consecutive cycles, whereas other participants only needed to ovulation test once. The final sample of 50 participants contributed a total of 152 visits (*n*_menstrual_ = 50; *n*_ovulatorry_ = 50; *n*_mid-luteal_ = 52). Two participants completed a total of 4 visits since their mid-luteal assessment had to be repeated due to an abnormal luteal phase length (>20 days). Thus, a total of 150 visits (*n*_menstrual_ = 50; *n*_ovulatorry_ = 50; *n*_mid-luteal_ = 50) were included in the cycle phasing process below.

#### 3.1.8. Cycle Phase Criteria

In contrast to study one, participants were aware of the menstrual cycle focus of the study, allowing them to assist the study team in scheduling the visits so that they would fall into specific cycle phases (mid-luteal, perimenstrual, ovulatory). As described above, the three visits were scheduled based on each participant’s onset of menses and positive LH-test result. Since LH-tests, like all measures, have limits, they should be used in conjunction with other sources of information to validate cycle phase. Therefore, after data collection was completed, each visit was characterized by (1) its forward- and backward count cycle day (see study one; Section 2.1.6), and (2) its E2 and P4 level. The following cycle phase criteria were applied:

1. Forward- and backward count cycle day. Ovulatory: cycle day −12 to −17; mid-luteal: cycle day −4 to −11; perimenstrual: perimenstrual: cycle day −3 to +3 (where day 1 is menstrual onset and there is no day 0).
2. Absolute hormone levels. The company supplying the antibody ELISA kits for the salivary hormone analyses (IBL; Hamburg, Germany) provided a salivary luteal cut-off for P4 of 127 pg/mL. Even though IBL stressed that this cut-off should not be the only reason for any therapeutical consequences, it served the present study as a first reference point and the following criteria: ovulatory: P4 < 127 pg/mL; mid-luteal: P4 ≥ 127 pg/mL; perimenstrual: - (note: Since E2 and P4 levels perimenstrually decline from their mid-luteal peaks, no absolute cut-off could be applied).
3. Relative hormone levels. Ovulatory: P4_ovulatory_ < P4_mid-luteal_; mid-luteal: P4 _mid-luteal_ > P4_ovulatory_ (note: Since P4 shows a singular peak (mid-luteal) while E2 has a primary (ovulatory) and a secondary (mid-luteal) peak, defining relative differences in hormone levels is more reliable regarding P4 than E2. For example, it cannot be said with absolute certainty that E2_ovulatory_ > E2_luteal_, since E2 peaks shortly before ovulation and then decreases relatively quickly, so that at the time of the positive ovulation test the already slightly decreased E2 levels (i.e., E2_ovulatory_) might already resemble E2_mid-luteal_); perimenstrual: E2_perimenstrual_ < E2_ovulatory_ and P4_perimenstrual_ < P4_mid-luteal_.

#### 3.1.9. (Re)categorizing Cycle Phases

The procedure for (re)assigning visits to a cycle phase for analysis was as follows: If a visit met both criterion 1 (forward- and backward count cycle day) and criterion 2 (absolute hormone levels) of its originally scheduled cycle phase, they remained in this cycle phase and were included in the analyses. This was the case with 87 of the total of 150 visits. If criteria 1 and/or 2 were not fulfilled, the visit was further consulted by two experts (K.M.S., T.A.E.-M.) and criterion 3 (relative hormone level) was taken into account: 20 of the 63 assessments not meeting criterion 1 and/or 2 were assigned to their originally scheduled cycle phase since the relative fluctuations of P4 and E2 (criterion 3) supported the original phase. In 5 cases, the assessments were reassigned to a different cycle phase than the one they were originally scheduled in since the relative levels of P4 and E2 (criterion 3) indicated that the visit was actually conducted in another cycle phase (originally ovulatory reassigned to mid-luteal: *n* = 2; originally mid-luteal reassigned to ovulatory: *n* = 1; originally mid-luteal reassigned to perimenstrual: *n* = 2). The remaining 38 visits which did not meet criterion 1 and/or 2 and did not fulfill criterion 3 were excluded from cycle phase analyses since they could not be confidently assigned to any of the three cycle phases of an ovulatory cycle. More detailed information is provided in the Supplementary Materials 2.

#### 3.1.10. Statistical Analyses

The same analysis approach as in study one (see Section 2.1.7) was also used for study two. The corresponding dataset for study two is provided in the Supplementary Materials 2.

### 3.2. Results

#### 3.2.1. Descriptive Analyses

For cycle phase analyses, a total of 112 visits from ovulatory cycles of 44 participants were included. Descriptive information for E2, P4 and HFlog levels are listed for each cycle phase separately in Table 4. In this study, E2 demonstrated a more pronounced peak in the ovulatory phase, consistent with our LH-surge-based method of scheduling ovulatory phase visits, which is more likely to include peak E2 levels relative to count-based methods. P4 demonstrated a clear peak in the midluteal phase. 

For hormonal analyses, a total of 150 visits from 50 participants were included. 

#### 3.2.2. Multilevel Regression Models Predicting HFlog from Menstrual Cycle Phase

Results of multilevel models examining pairwise comparisons of HFlog between each cycle phase are presented in Table 5. Hypothesis 1 predicted HRV to be lower in the midluteal phase relative to both the mid-follicular and ovulatory phases. Since study two did not assess mid-follicular HRV levels, only the midluteal and ovulatory phases could be compared to test this hypothesis. However, the hypothesis could not be confirmed as mid-luteal HFlog was not significantly different from ovulatory HFlog. Only the comparison of the mid-luteal and perimenstrual phase reached significance.

#### 3.2.3. Multilevel Regression Models Predicting HFlog from E2 and P4

Results of the model predicting HFlog from E2, P4, and their interaction are reported in Table 6. A random slope was observed for P4, indicating significant individual differences in the degree to which changes in P4 were associated with changes in HFlog. As hypothesized, fixed effects indicated that only P4 was significantly associated with (lower) HFlog. In line with study one, hypotheses 2 and 3 could thus be confirmed in study two as well. Of note, this result is consistent with the finding that the midluteal phase, which is characterized by a peak in P4, shows the lowest HFlog.

## 4. General Discussion

In a recent meta-analysis of 37 studies and 1004 participants, we reported a significant decrease in cardiac vagal activity from the follicular to the luteal phase [28]. However, the question remained which of the two ovarian hormones (E2, P4, or the interaction of both) is associated with these cyclical HRV changes. Here, we presented two longitudinal studies on the unique roles of E2 and P4 in menstrual cycle-related changes in HRV. The two studies share their repeated assessment of ovarian hormones and HRV in naturally-cycling participants while they differ in various design aspects (most prominently, that study one assessed the effects of hormones on HRV regardless of cycle phase, while study two assessed these effects at very specific cycle phases). As the focus of the present work is on ovarian hormones (as opposed to cycle phase, given that recent meta-analytic results are already available on this topic [28]), these design differences allow for testing the effects of the hormones themselves and the robustness of these effects.

In line with the results of our recent meta-analysis [28], study one found reduced HRV in the midluteal phase relative to the mid-follicular phase. Analyses in study one, which used backward-counting (a less precise method for identifying the ovulatory phase), also revealed that the ovulatory phase was associated with higher HRV than the midluteal phase. However, in study two, which scheduled participants for ovulatory visits only following a positive urine LH test, we did not find a difference in HRV between the ovulatory and midluteal phases. This may be because this more precise measure led to consistently post-ovulatory visits in study two, during which P4 is already rising and potentially exerting effects on HRV, which may therefore diminish the contrast between the ovulatory and midluteal phases in study two. Also, both studies revealed significantly lower HRV levels in the mid-luteal compared to the perimenstrual phase. Therefore, our data are consistent with our first hypothesis, that the midluteal phase is associated with a general reduction in HRV.

The goal of the present paper was to test (and conceptually replicate) the within-person associations of endogenous E2 and P4 with HRV. Consistent with our hypotheses, and with the midluteal phase reduction in HRV, within-person fluctuations in P4 (and not E2 or their interaction) were significantly associated with (lower) HRV in both studies. Of note, both studies also found significant random slopes for the influence of P4 on HRV, indicating possible individual differences in these associations that should be explored further in larger samples. Our confidence in the validity of these associations is bolstered by our ability to conceptually replicate these P4-HRV associations across two studies with differing methods.

### 4.1. Possible Underlying Mechanisms

Our recent meta-analysis demonstrated a significant decrease in cardiac vagal activity from the follicular to luteal phase [28]. The results of the present work suggest that it is likely high levels of P4 (rather than P4 withdrawal) that lead to decreased midluteal HRV. Although the observational study design of the present studies (as opposed to experiments) does not allow for firm conclusions about causal underlying mechanisms, several possible pathways are discussed below.

The ANS is composed of the peripheral sympathetic and parasympathetic branches and the central autonomic network (i.e., a network of brain areas involved in autonomic control; CAN), which jointly regulate HRV. As reviewed by Thayer and colleagues [4,12,13], the CAN includes the prefrontal, cingulate, and insula cortices and the amygdala. Animal research on ovarian hormones has revealed that P4 receptors are broadly expressed throughout the brain, including CAN areas important for HRV regulation like the amygdala and the frontal cortex [46,47,48]. It is therefore possible that menstrual cycle-related changes in P4 may cause HRV fluctuations by acting in certain CAN areas. 

In general, there are two primary central mechanisms by which P4 can act: (1) ‘classical’ P4 receptor-mediated pathways (genomic effects), and (2) alternate ‘non-genomic’ mechanisms [46]. These non-genomic mechanisms generally refer to the metabolism of P4 to neuroactive steroids, of which allopregnanolone (ALLO) and pregnanolone are among the two most studied [48]. In naturally-cycling individuals, plasma levels of ALLO reach approximately 0.2–0.5 nmol/L in the follicular and up to 4 nmol/L in the luteal menstrual cycle phase [49]. ALLO modulates the GABA system by acting as a positive allosteric modulator of GABA-A receptors. The common effects of ALLO and other positive modulators of GABA-A receptor (e.g., alcohol, benzodiazepines, barbiturates) are inhibitory (e.g., anesthetic, sedative, anticonvulsant, anxiolytic) [49,50]. Research on the effects of alcohol, another positive allosteric modulator of the GABA-A receptor, on HRV indicates that acute alcohol ingestion quickly reduces HRV [51]. Further, alcohol-induced reductions in HRV are thought to be mediated by the GABA system [52], and experimental animal data suggests that alcohol modulates GABA-mediated cardiovascular control mechanisms in the brainstem [53]. Future studies should investigate whether these two modulators of the GABA-A receptor (alcohol and ALLO) effect HRV via similar mechanisms. 

In addition to the anesthetic, sedative, anticonvulsant, and anxiolytic effects of P4 metabolites such as ALLO, numerous studies have shown that the effects of the positive modulators of GABA-A receptor can also be exactly the opposite (i.e., paradoxical). These paradoxical effects (which can take the form of negative mood, anxiety, irritability or aggression) severely affect 3 to 6% of human subjects and moderately affect 25% [49,50]. Research on the emotional effects of the menstrual cycle suggests that affective symptoms experienced in the luteal phase of the menstrual cycle by individuals with premenstrual dysphoric disorder (PMDD) may be caused by the paradoxical effects of ALLO mediated by the GABA-A receptor [49]. Thus, individuals seem to differ in the extent to which paradoxical effects of ALLO cause affective symptoms in the luteal phase. Future research should investigate whether, (and if so, for whom) paradoxical effects of ALLO might also serve as an underlying mechanism for P4-related HRV reduction in the luteal phase.

Given that the amygdala (which is involved in HRV regulation as part of the CAN [13]) is rich in GABA-A receptors [54], possible P4-induced changes in the activity of the amygdala (mediated by ALLO as a positive modulator of GABA-A receptors) are of interest. However, the literature is conflicting regarding a potential effect of P4 on HRV via the amygdala. Van Wingen and colleagues combined a single progesterone administration to naturally-cycling individuals with functional magnetic resonance imaging (fMRI) in two double-blind, placebo-controlled, crossover design studies [55,56]. Participants received the P4 administration in their follicular phase which yielded plasma concentrations of P4 and ALLO comparable to levels observed during pregnancy in one study [55] and comparable to luteal phase levels in the other study [56]. Interestingly, moderate (i.e., luteal phase) ALLO plasma concentrations increased amygdala reactivity (to angry and fearful faces) [56], while higher (i.e., pregnancy) ALLO plasma concentrations decreased the amygdala reactivity [55], indicating that ALLO may modulate amygdala activity in a biphasic dose-dependent manner. A (somewhat conflicting) study using positron emission tomography (PET) revealed a positive correlation between amygdala activity and HRV in females [57]. Since the amygdala is a common neural basis for both P4 and HRV functioning, future studies should continue to examine effects of P4 in the amygdala as a possible mechanism for the P4-HRV correlation. 

In sum, much more research is needed to understand the underlying mechanisms of the effects of cycle-related fluctuations of P4 on the regulation of HRV. This is in line with the conclusion of a recent review of research on cardiovascular disease in women, which stated that the role of P4 in the regulation of cardiovascular physiology remains largely unknown and requires further research [47].

### 4.2. Clinical Implications

Regarding clinical implications, the results of the present work might be relevant for the field of cardiology: Given that HRV has established itself as a biomarker for cardiac health in research [9], recent studies suggest its use as a tool for cardiac risk assessment in clinical practice [8,58]. If clinicians were to use HRV assessments for clinical reasons, they should keep the within-person fluctuations of HRV in naturally-cycling females in mind. We recommend mid-follicular assessments with low and stable P4 levels (i.e., between day +5 and +10, where the onset of menses is day +1 and there is no day 0). 

Given HRV’s association with cognitive and emotional self-regulatory capacity [1,2,3,4,5], the results of the present work could also have implications for everyday functioning. However, since no clinical outcomes (e.g., affective and cognitive symptoms) were reported in the present work, it cannot be safely concluded that the observed fluctuations in HRV across the menstrual cycle are associated with fluctuations in clinical symptoms. As will be stated below in the section on future research (see Section 4.3), this should be investigated in future studies.

### 4.3. Implications for Future Research

The association of cyclical P4 with vagally-mediated HRV has several important implications for future research: First, future studies on cardiac vagal activity (indicated for example by HRV) as a predictor or outcome should control the cycle phase of the naturally-cycling participants. Cycle phase should either be included in the analyses as a covariate or, preferably, assessments should take place in the same cycle phase for all cycling individuals. A failure to take the menstrual cycle of female participants into account in HRV studies could greatly reduce the significance and validity of findings. The same implication was already found in the meta-analysis showing significant differences in cardiac vagal activity across menstrual cycle phases [28]. However, the present work can empirically specify this implication based on its result that it is high levels (as opposed to withdrawing levels) of the ovarian hormone P4 (and not E2), which are related to the menstrual fluctuations of cardiac vagal activity (indicated by HRV). We therefore specifically recommend future studies on cardiac vagal activity to schedule assessments in the mid-follicular phase of their naturally-cycling participants since this phase is characterized by low and stable P4 levels. Assessments in this cycle phase are practicable to schedule, as it is possible to count forwards from the more clearly reportable onset of menses and set assessments between days +5 to +10.

A second implication for future research is based on the above-mentioned empirical evidence for the links between HRV and cognitive functioning [4], emotional functioning [1,3,5] and behavioral outcomes [2]. These results mainly stem from cross-sectional studies and therefore reveal inter-individual differences in cognitive, affective and behavioral outcomes as a function of trait-level HRV. The results of the present work imply the need for studies investigating whether the intra-individual fluctuations in HRV across the menstrual cycle are paralleled by intra-individual cycle-related variations in cognitive, affective and behavioral outcomes across the cycle. There may also be differences as to which outcome (affective, cognitive or behavioral) is more closely associated to the HRV fluctuations across the menstrual cycle. Given the present work’s result that menstrual HRV fluctuations are related to the ovarian hormone P4 (and not E2), future studies can plan their repeated assessments of these outcomes in different cycle phases accordingly: Assessments in the mid-luteal phase with its high P4 levels and control assessments, for example, in the mid-follicular phase with its stable low P4 levels, should be performed.

The third implication for future research is based on previous empirical work on the emergence of clinically significant emotional, cognitive and physical symptoms in the premenstrual phase and subsequent clearance of symptoms in the follicular phase affecting a minority of cycling individuals (i.e., the new DSM-5 diagnosis PMDD [17]). Experimental studies demonstrate that PMDD is not characterized by abnormal fluctuations or levels of E2 and P4; rather, it is caused by abnormal responses to normal fluctuations and levels that cause these symptoms [23]. Individuals affected by PMDD are therefore ascribed *hormone sensitivity* [59]. Previous research traced inter-individual differences in hormone sensitivity (indicated by the emergence of PMDD symptoms) back to, for example, traumatic experiences in the woman’s biography [60,61,62,63]. In the light of the present work, the question is whether intra-individual fluctuations of HRV across the cycle may be a physiological marker of hormone sensitivity (comparable to traumatic experiences as a biographical marker). Cyclical fluctuations of HRV as a biomarker for hormone sensitivity would take research on female health an important step further.

### 4.4. Strengths and Limitations

When considering the work as a whole, the primary methodological strength lies in the repeated measurement design investigating intra-individual variations in HRV across the menstrual cycle as opposed to cross-sectional study designs which do not do justice to the inter-individual differences in cycle effects. However, even though analyses in both studies revealed random slopes for the effect of P4 on HRV (i.e., inter-individual differences regarding menstrual cycle-related variations in HRV), there was not enough power in either study to investigate the between-person predictors of these differences in hormone sensitivity further, and this is a central limitation of the studies.

As mentioned before, the two studies included in the present work show various design differences. However, given that the focus of the present work is the association between cyclical ovarian hormones and HRV, the most central variables of interest (i.e., E2, P4, and HRV) were measured with a high degree of consistency between studies: In both studies, saliva samples were used to analyze hormone levels, the same ECG lead pattern was used, participants were seated during their ECG, high frequency RSA was employed as an index for vagally-mediated HRV, statistical analytic methods were identical, and participants were naturally-cycling females of similar age. The two studies are therefore compatible with each other with regard to the question of how the ovarian hormones are associated with HRV. Also the same results (high levels of P4 are associated with low levels of HRV) are yielded in both studies, indicating that the effects of P4 are robust to changes in study design. Given the replication crisis in psychological research, the methodological differences of the two studies could therefore be perceived as an advantage and not a disadvantage [38].

One central design difference between the studies was the disguise of the menstrual cycle focus in study one but not in study two. On the one hand, this blinding allowed the assessment of the association between ovarian hormones and HRV in study one to be less affected by cycle-related stereotypes and expectations than in study two. On the other hand, the blinding reduced the precision of cycle phase estimations as data points in study one had to be phased in retrospect (via the cycle day count-based method). As a result, 55 of the 160 assessments had to be excluded in the cycle phase analyses of study one since they did not fall within any cycle phase time window and the ovulatory E2 concentrations did not show the typical exponential increase. Most likely, the cycle day count-based method of determining the ovulatory cycle phase included both peak E2 days as well as lower-E2 peri-ovulatory days. However, this study design allowed for testing the primary question of the present work as it allowed for clear examination of the association of hormones (and less cycle phase) and HRV within a given individual across the cycle. Another difference between the two studies refers to the ECG data lengths (5 min in study one vs. 10 min in study two). However, as the present work investigates within-person changes in HRV, and since the ECG data lengths were consistent within each participant, different ECG data lengths in the two studies are defensible. Also, separate statistical models were analyzed for each study resulting in consistent ECG data lengths within each statistical model.

A limitation shared by both studies refers to the time of day of HRV assessments. Given that HRV shows circadian variations [64,65] and since the present work focuses on within-person changes in HRV, we have kept the time of the testing consistent within each participant. Nonetheless, it is possible that the time between waking and testing showed within-person differences which, in turn, could distort the investigated effects of cyclical hormones on HRV. Unfortunately, we failed to collect information on the time that has passed between waking up and testing, which is a limitation of both studies.

Another limitation specific to study two refers to the reduced reliability of the ovulation tests used: Since the tests exhibited a high sensitivity to low LH levels, it is possible that they generated false-positive results in participants with a relatively high baseline LH level. In addition, the comparison of the test and control lines was often ambiguous, making the read-out susceptible to errors. For this reason, the cycle phase reclassification process included the backward-count day approach. This approach, however, does less justice to inter-individual differences in cycle lengths than does ovulation testing.

Finally, since the two studies were observational studies investigating concurrent fluctuations in ovarian hormones and HRV across the menstrual cycle, it is not possible to refer to causal cycle effects which is another central limitation of this work.

## 5. Conclusions

Numerous studies have linked vagally-mediated HRV to psychological outcomes (such as cognitive and emotional self-regulatory capacity [1,2,3,4,5]) and physical factors (such as cardiac risk [6,7,8,9,10]), resulting in the emergence of HRV as a biomarker for physical and mental health [11,12,13]. Understanding the factors that give rise to intra-individual changes in vagally-mediated HRV is therefore critical. Our recent meta-analysis [28] revealed that cardiac vagal activity (most commonly indicated by vagally-mediated HRV) decreases significantly from the follicular to the luteal cycle phase. However, since the meta-analysis [28] used cycle phase as a proxy for ovarian hormone levels, the question remained (till the current studies) which ovarian hormone, E2, P4 or the interaction of both, is more strongly related to the HRV fluctuations. The present work reports on findings of the first two studies using repeated measures of ovarian hormones and HRV across the menstrual cycle to model both the unique and interactive effects in person-centered E2 and P4 on HRV in multilevel models (with phase observations nested within woman). Despite differences in the study design, both studies revealed a significant negative association between P4 and vagally-mediated HRV, indicating the robustness of this result. No significant main or interactive effects of E2 on HRV were found. Cycle phase comparisons in both studies were consistent with these results with lower HRV levels in the mid-luteal phase (which is characterized by high P4 levels) than in other phases, which also replicates the findings of the meta-analysis [28]. Future female health studies should investigate inter-individual differences in these effects of P4 on HRV (e.g., as a function of fitness level or hormone sensitivity), and whether the conjoint cycle-related fluctuations of vagally-mediated HRV and P4 are accompanied by fluctuations in daily functioning. Experimental studies should also explore the causal underlying mechanisms of the negative HRV-P4 association. In studies that focus on cardiac vagal activity, and where the influence of the menstrual cycle should not distort the associations in question, naturally-cycling female participants should always be investigated in their mid-follicular phase (which is characterized by low and stable P4 levels). The same applies to the clinical setting if vagally-mediated HRV was assessed in naturally-cycling individuals as an indicator for cardiac risk.

## Figures and Tables

**Table 1:** Mean and Standard Deviation of Estradiol (E2), Progesterone (P4) and Log High Frequency Heart Rate Variability (HFlog) across the Menstrual Cycle Phases Investigated in Study One (*N* = 105 visits).

Menstrual Cycle Phase	E2	P4	HFlog
Midluteal	3.52 (0.73)	14.87 (8.81)	5.61 (1.06)
Perimenstrual	3.14 (0.86)	9.97 (5.12)	5.89 (1.05)
Mid-follicular	3.40 (1.22)	8.10 (3.95)	6.15 (1.06)
Ovulatory	3.85 (0.88)	7.13 (4.36)	6.01 (1.09)

Note. Estradiol is shown in pg/mL. Progesterone is shown in ng/dL.

**Table 2:** Multilevel Regression Models Predicting Log High Frequency Heart Rate Variability (HFlog) from Menstrual Cycle Phase (Within-Person Contrasts) in Study One (*N* = 105 visits).

Within-Person Contrasts					
Midluteal Phase Reference			Perimenstrual Phase Reference		Mid-Follicular Phase Reference
**v. Mid-follicular**	**v. Ovulatory**	**v. Peri- menstrual**	**v. Ovulatory**	**v. Follicular**	**v. Ovulatory**
0.55* (0.24)	0.60* (0.29)	0.52* (0.23)	0.08 (.21)	0.03 (0.15)	0.04 (0.21)

Note. * *p* < 0.05. Significant parameters are shown in **bold**. Positive values indicate higher levels of HRV in the comparator phase relative to the reference phase.

**Table 3:** Multilevel Regression Models Predicting Log High Frequency Heart Rate Variability (HFlog) from Estradiol (E2) and Progesterone (P4) in Study One (*N* = 158 visits).

Parameter	Outcome: HFlog (Vagally-Mediated Heart Rate Variability)	
	Estimate	SE
**Fixed Effects**		
Intercept (*γ_00_*)	5.98	0.16
Sample-Standardized Age (*γ*_01_)	0.06	0.15
Person-Centered E2 (*γ*_10_)	−0.037	0.085
Person-Centered P4 (*γ*_20_)	−0.036 ***	0.011
E2 * P4 (*γ*_30_)	−0.015	0.025
**Variance Components**		
Intercept (*u*_0__*j*_)	0.86	0.22
Person-Centered P4 (*u*_2__*j*_)	0.016 *	0.0076
Autoregressive (visit-1) Term	−0.14	0.14
Residual (*e_ij_*)	0.30	0.043

Note. * p < 0.05, *** *p* < 0.001. Statistically significant parameters are shown in **bold**.

**Table 4:** Mean and Standard Deviation of Estradiol (E2), Progesterone (P4) and Log High Frequency Heart Rate Variability (HFlog) across the Menstrual Cycle Phases Investigated in Study Two (*N* = 112 visits).

Menstrual Cycle Phase	E2	P4	HFlog
Midluteal	4.79 (2.70)	17.95 (7.98)	5.94 (1.42)
Perimenstrual	3.43 (2.62)	5.78 (3.22)	6.39 (1.17)
Ovulatory	6.01 (2.94)	6.13 (2.97)	6.12 (1.06)

Note. E2 is shown in pg/mL. P4 is shown in ng/dL.

**Table 5:** Multilevel Regression Models Predicting Log High Frequency Heart Rate Variability (HFlog) from Menstrual Cycle Phase (Within-Person Contrasts) in Study Two (*N* = 112 visits).

Within-Person Phase Contrast		
Midluteal Phase Reference		Perimenstrual Phase Reference
v. Ovulatory	v. Perimenstrual	v. Ovulatory
0.17 (0.17)	0.45 * (0.17)	−0.28 (0.17)

Note. * *p* < 0.05. Significant parameters are shown in **bold**. Positive values indicate higher levels of HRV in the comparator phase relative to the reference phase.

**Table 6:** Multilevel Regression Models Predicting Log High Frequency Heart Rate Variability (HFlog) from Estradiol (E2) and Progesterone (P4) in Study Two (*N*=150 visits).

Parameter	Outcome: HFlog (Vagally-Mediated Heart Rate Variability)	
	Estimate	SE
**Fixed Effects**		
Intercept (*γ_00_*)	6.21	0.18
Sample-Standardized Age (*γ*_01_)	−0.23	0.17
Person-Centered E2 (*γ*_10_)	−0.005	0.037
Person-Centered P4 (*γ*_20_)	−0.024 *	0.010
E2 * P4 (*γ*_30_)	0.003	0.008
**Variance Components**		
Intercept (*u*_0__*j*_)	1.12	0.33
Person-Centered P4 (*u*_2__*j*_)	0.02 *	0.009
Autoregressive (visit-1) Term	0.19	0.26
Residual (*e_ij_*)	0.58	0.17

Note. * p < 0.05. Statistically significant parameters are shown in **bold**.

## Supplementary Materials

The following are available online at https://www.mdpi.com/2077-0383/9/3/617/s1, Supplementary Materials 1: Dataset Study One; Supplementary Materials 2: Dataset Study Two.
